# Dopamine-Related Reduction of Semantic Spreading Activation in Patients With Parkinson’s Disease

**DOI:** 10.3389/fnhum.2022.837122

**Published:** 2022-03-31

**Authors:** Hannes Ole Tiedt, Felicitas Ehlen, Fabian Klostermann

**Affiliations:** ^1^Department of Neurology, Motor and Cognition Group, Charité—Universitätsmedizin Berlin, Corporate Member of Freie Universität Berlin and Humboldt-Universitätzu Berlin, Berlin, Germany; ^2^Department of Psychiatry, Jüdisches Krankenhaus Berlin, Berlin, Germany; ^3^Berlin School of Mind and Brain, Humboldt-Universität zu Berlin, Berlin, Germany

**Keywords:** Parkinson’s disease, dopamine, semantic system, spreading activation, verbal fluency

## Abstract

Impaired performance in verbal fluency (VF) tasks is a frequent observation in Parkinson’s disease (PD). As to the nature of the underlying cognitive deficit, it is commonly attributed to a frontal-type dysexecutive syndrome due to nigrostriatal dopamine depletion. Whereas dopaminergic medication typically improves VF performance in PD, e.g., by ameliorating impaired lexical switching, its effect on semantic network activation is unclear. Data from priming studies suggest that dopamine causes a faster decay of semantic activation spread. The aim of the current study was to examine the impact of dopaminergic medication on the dynamic change of word frequency during VF performance as a measure of semantic spreading activation. To this end, we performed a median split analysis of word frequency during phonemic and semantic VF task performance in a PD group tested while receiving dopaminergic medication (ON) as well as after drug withdrawal (i.e., OFF), and in a sample of age-matched healthy volunteers (both groups *n* = 26). Dopaminergic medication in the PD group significantly affected phonemic VF with improved word production as well as increased error-rates. The expected decrease of word frequency during VF task performance was significantly smaller in the PD group ON medication than in healthy volunteers across semantic and phonemic VF. No significant group-difference emerged between controls and the PD group in the OFF condition. The comparison between both treatment conditions within the PD group did not reach statistical significance. The observed pattern of results indicates a faster decay of semantic network activation during lexical access in PD patients on dopaminergic medication. In view of improved word generation, this finding is consistent with a concept of more focused neural activity by an increased signal-to-noise ratio due to dopaminergic neuromodulation. However, the effect of dopaminergic stimulation on VF output suggests a trade-off between these beneficial effects and increased error-rates.

## Introduction

Cognitive symptoms are frequently observed in patients with Parkinson’s disease (PD) throughout all stages of the disease, ranging from subtle cognitive changes to mild cognitive impairment and dementia (Cooper et al., 1991; Aarsland et al., 2003; Stefanova et al., 2015). As to the nature of these impairments, cognitive abnormalities associated with PD are most often characterized as a frontal-type dysexecutive syndrome due to fronto-striatal dopamine depletion (Lange et al., 1992; Owen et al., 1992; Taylor and Saint-Cyr, 1995; Cools et al., 2001b, 2003; Lewis et al., 2003; Kübler et al., 2017; Chung et al., 2018; for reviews see Kehagia et al., 2010; Robbins and Cools, 2014). The effect of dopaminergic medication on cognitive symptoms in PD, however, is ambiguous ranging from improvement of certain impairments to negative effects on other functions at the same time (e.g., Cools et al., 2001a, 2003). It was suggested, that such dissociated effects of increased dopaminergic transmission are due to the restoration of fronto-striatal dopamine depletion combined with the overdosing of less depleted dopaminergic circuits at the meso-cortical (prefrontal) level (Cools et al., 2001a, see also Robbins and Cools, 2014).

In addition, a more heterogeneous picture of cognition in PD has emerged, as other cognitive domains may be impaired in PD in addition to executive functions: this includes impairments in semantic processing (MacDonald et al., 2019; Stögbauer et al., 2020), as well as a variety of language functions including pragmatic abilities such as metaphor comprehension (Monetta and Pell, 2007; Baraldi et al., 2021), syntactic processing (Johari et al., 2019), verb use (Holtgraves et al., 2010; Salmazo-Silva et al., 2017), and lexico-semantic retrieval (Foster et al., 2008; Salmazo-Silva et al., 2017; Silveri et al., 2018; Wagner et al., 2020). The most consistent finding with respect to language functions, however, is a significantly reduced verbal fluency (VF) in PD patients (e.g., Gotham et al., 1988; Raskin et al., 1992; Flowers et al., 1995; Troyer et al., 1998; Obeso et al., 2012; El-Nazer et al., 2019). Although originally considered as tests of primarily frontal-lobe functions based on early clinical findings (e.g., Milner, 1964; Benton, 1968), converging evidence rather supports a perspective on VF tasks as a multimodal testing procedure including executive functions such as inhibition of inappropriate responses and self-monitoring as well as semantic and language-related processing functions of a fronto-temporal network (Phillips, 1997; Ruff et al., 1997; Hirshorn and Thompson-Schill, 2006; Birn et al., 2010; Clark et al., 2014; Glikmann-Johnston et al., 2015; Whiteside et al., 2016; Li et al., 2017; see also Ralph et al., 2017). In this view, reduced VF output in PD patients appears consistent with the more widespread linguistic deficits observed in other studies.

Concerning the different cognitive operations underlying VF task performance, impaired word generation during VF tasks in PD patients has been most consistently associated with impaired conceptual switching between lexical fields during word search and retrieval (Troyer et al., 1998; Donovan et al., 1999; Farzanfar et al., 2018). Both dopaminergic medication and Deep Brain Stimulation of the subthalamic nucleus (STN-DBS) led to improved lexical switching when tested ON as compared with OFF treatment (Vonberg et al., 2016; Tiedt et al., 2020). With respect to automatic spreading activation in lexical fields within a conceptual network as reflected by word-clusters (Troyer et al., 1997), both treatment strategies produced slightly differential effects: whereas dopaminergic medication led to the formation of smaller clusters in pharmacologically treated PD patients (Tiedt et al., 2020), there was no significant change of cluster size by STN-DBS (Vonberg et al., 2016).

Further evidence for a specific dopaminergic modulation of automatic spreading activation within semantic networks in addition to effects on executive functions can be derived from results of word priming studies. Both priming and word search during VF tasks are, from a conceptual perspective, similar with respect to a supposed underlying automatic spreading activity within semantic networks (Collins and Loftus, 1975). In this view, long-term memory representations are organized in larger networks (e.g., “duck” is contained in the category “birds” or, at a larger scale in “animals”) and each “node” within the network contains the semantic knowledge related to an item. Each node is bi-directionally connected with other related nodes, and the strength of these links is determined by the degree of internodal semantic association as well as the frequency of nodal activation (Allen et al., 1992).

Upon accessing the lexical representation of a given concept, semantically related nodes in this network become pre-activated through a spreading activation along the connections between associated concepts and words. The corresponding facilitating of word retrieval has, e.g., been demonstrated in classical priming experiments (Meyer and Schvaneveldt, 1971). Of note, this mechanism of spreading activation during lexical retrieval has been associated both with word recognition and speech production (e.g., Roelofs, 1992; Dell et al., 1997).

With respect to the influence of dopaminergic neuromodulation, indirect word priming, which is thought to require more widespread or sustained semantic activation, was selectively disrupted by dopamine administration in healthy volunteers (Kischka et al., 1996; Angwin et al., 2003, 2004; Roesch-Ely et al., 2006). Supporting this evidence, a similar effect was observed in PD patients receiving dopaminergic medication (Angwin et al., 2006, 2007; Arnott et al., 2011). These findings were explained with a non-specific neuromodulatory role of dopaminergic neurons (Le Moal and Simon, 1991), leading to the suppression of weaker signals in favor of stronger ones through a kind of “gating mechanism” (Servan-Schreiber et al., 1990; Cepeda and Levine, 1998). In terms of the spreading activation theory of semantic processing as posited by Collins and Loftus (1975), increased dopaminergic transmission should be associated with an earlier or faster decay of the activity spread along bi-directionally connected nodes within the semantic network (Kischka et al., 1996; Angwin et al., 2004). However, in view of potential changes of semantic activation in PD, some authors have concluded that dopamine depletion might result in delayed, i.e., a slower rise and fall of activity (Arnott et al., 2001), or even increased (Foster et al., 2008) semantic spreading activation. Therefore, the question remains how both dopamine depletion and, on the other hand, pharmacological therapy contribute to a suggested alteration of semantic activation in individuals with PD.

**Figure 1:**
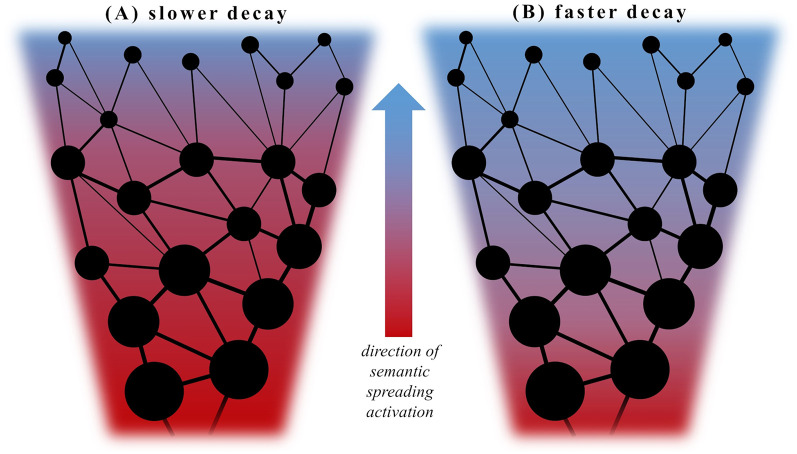
Decayof semantic spreading activation. Panels **(A)** and **(B)** show each a schematized segment of a conceptual network (or semantic category in terms of VF task performance) with densely interconnected stored items as “nodes”. Thick (high relatedness) or thin (lower relatedness) lines indicate the degree of semantic association between items. Larger points represent items with a high frequency of occurrence whereas smaller points infrequent ones. Semantic spreading activation is depicted as a centripetal flow from highly connected and frequent items produced early during VF tasks to more sparsely inter-connected and infrequent items in the periphery. The direction (or decay in time) of semantic spreading activation is indicated by the color shift from red to blue. In this view, relatively infrequent items require a stronger degree of (or more sustained) semantic activation for retrieval than more frequent ones. The retrieval of such low frequency items would therefore be facilitated by a slow decay **(A)** and impeded in case of faster decaying activation spread **(B)**. In the latter case, more highly frequent items would be accessed, possibly with a higher rate of repetition errors.

A possibility to address this issue with respect to semantic activation underlying word retrieval might emerge from investigating the effect of the frequency of use (i.e., how often a given word is encountered in language) in this context. Manipulations of word frequency have been shown to unfold consistent effects in studies of language processing, i.e., often used words are faster and more easily produced and recognized than infrequent words (Oldfield and Wingfield, 1965; Forster and Chambers, 1973; Allen et al., 1992; Jescheniak and Levelt, 1994; Morrison and Ellis, 1995; Gerhand and Barry, 1999; Brysbaert et al., 2000; Bonin and Fayol, 2002). Furthermore, the analysis of VF word output with respect to lexical frequency has demonstrated a characteristic dynamic change with a decrease of word frequency throughout task performance in both semantic and phonemic VF (Crowe, 1998; Juhasz et al., 2012; Zabberoni et al., 2017). With respect to the semantic spreading activation theory and lexico-semantic retrieval, lower-frequency words can be interpreted as being represented in more remote nodes within conceptual networks. These should require a more sustained and widespread semantic spreading activation than words with a higher frequency of use and are thus retrieved later during task performance (Foster et al., 2008). In this view, the suggested dopamine-induced increase of the signal-to-noise ratio would lead to the production of more “overlearned” responses and strengthen more direct and explicit associations between concepts (Kischka et al., 1996), thus favoring the retrieval of more high-frequency words. Furthermore, a faster decay of semantic activation following dopamine administration would predict a reduced decrease of word frequency throughout VF task performance as words with a lower frequency of use become less likely to be retrieved; see Figure 1. That being said, also an increased or delayed semantic activation (with a slower rise and dissipation of activity) is expected to cause a similar pattern, as more remote nodes may not be reached within the given amount of time. To be able to differentiate these hypothesized changes of semantic activation in patients with PD, we included healthy volunteers as controls and a PD group both following withdrawal of dopaminergic medication (i.e., OFF), and after receiving their regular pharmacological treatment (i.e., ON) in two separate sessions. We hypothesized two possible outcomes: (i) if dopamine depletion caused aberrant (i.e., delayed or increased) semantic activation in persons with PD, this would most likely result in a different pattern of activity in the PD group OFF medication relative to controls. This difference should become less clear or even disappear in the ON condition. (ii) If semantic activation is instead not significantly modulated by dopamine depletion, dopaminergic medication should unfold similar effects in persons with PD as in healthy controls in previous studies using word priming. In this case, we would expect an accelerated decay of semantic activation indexed by a smaller decrease of word frequency in the PD group ON medication and a similar pattern of semantic activation in healthy volunteers and the PD group in the OFF condition. To address this question, we planned to analyze the individual word output obtained in semantic and phonemic VF tasks with respect to its frequency of use as an index of semantic activation as well as standard VF parameters such as task performance and accuracy. The results will be discussed in light of earlier studies addressing the question of altered semantic activation in PD, which have yielded overall inconsistent results.

## Methods

### Participants

We studied data obtained from 26 individuals with PD, who participated in two experimental sessions while being on their regular medication (i.e., ON) as well as after withdrawal of all dopaminergic medication (i.e., OFF). The PD group in both medication conditions was compared to a group of 26 volunteers without any neurological or psychiatric conditions or relevant medication as controls. A part of this sample was included in a previously published analysis of clusters and switches (Tiedt et al., 2020). All participants (PD group and healthy volunteers) were recruited at the Department of Neurology of the Charité Universitätsmedizin—Berlin, Campus Benjamin Franklin (CBF). The order of the two experimental sessions in the PD group (ON and OFF medication) was changed within the PD group attempting to obtain a balanced order. Altogether, nine patients performed the experiment while being ON medication first compared with 17 individuals, which were OFF their medication during the first session. This uneven distribution was due to a higher number of patients not sustaining withdrawal of medication. The mean interval between both experimental sessions was 74 (± 41) days with a range of 32–175 days. Evaluations in the OFF condition followed a washout phase of at least 12 h for levodopa (L-3,4-Dihydroxyphenylalanin), and more than 24 h for dopamine agonists or sustained release preparations. The mean intervals (± S.D.) between the experimental session and the intake of medication were 1.8 (± 1.6) h in the ON condition and 19.4 (± 5.2) h in the OFF condition. The levodopa-equivalent daily dose (LEDD) for each individual was calculated according to Tomlinson et al. (2010); for the group mean LEDD see Table 1. The motor scale (part III) of the Unified Parkinson’s Disease Rating Scale (UPDRS; Fahn and Elton, 1987) was assessed during the ON and OFF conditions by an experienced clinician. All participants completed the Parkinson Neuropsychometric Dementia Assessment (PANDA) as a screening test for dementia, using a cut-off value of <14 points as an exclusion criterion (Kalbe et al., 2008). Prevalence of psychiatric symptoms was assessed based on a standardized rating of psychopathological symptoms (AMDP, 2016), any relevant current psychiatric comorbidity (e.g., depression, psychotic features, obsessive-compulsive symptoms) were exclusion criteria for study participation. These inclusion criteria were applied to both the clinical and control group.

**Table 1:** Sample characteristics.

	Controls	PD group
Age mean [years]	67.1 (± 7.5)	70.0 (± 8.9)
Education mean [years]	10.8 (± 2)	11.2 (± 1.6)
Gender: female/male	9/17	12/14
Handedness: right/left	22/4	25/1
total PANDA score	24.4 (± 4.1)	23.5 (± 4.4)
PANDA range	14–30	16–30
Disease duration [years]		6.7 (± 5.8)
Side of onset: right/bilateral/left		16/2/8
UPDRS III on		19.4 (± 12.8)
UPDRS III off		28.1 (± 11.0)
UPDRS III difference		8.8 (± 10.5)
LEDD [mg]		751 (± 417)

*Demographic variables of both groups and clinical characteristics of the PD group in both ON and OFF conditions as noted. All values are the Mean (± S.D.). Abbreviations: PANDA, Parkinson Neuropsychometric Dementia Assessment; UPDRS, Unified Parkinson’s Disease Rating Scale; LEDD, Levodopa Equivalent Daily Dose*.

Both participant groups were compared by using the χ^2^-test for dichotomous data (gender, handedness) and independent two-sample *t*-tests or Mann-Whitney *U* tests (depending on the normal distribution of the data) for non-dichotomous data (age, education, PANDA scores). Within-group comparisons of non-dichotomous data (UPDRS III scores) were conducted using paired-samples *t*-tests or Wilcoxon-tests. An overview of the sample characteristics can be found in Table 1. The experiment was evaluated and approved by the institutional ethics committee of the Charité—Universitätsmedizin Berlin (EA2/047/10). All participants gave their informed and written consent prior to the experiments, and all reported research was conducted in accordance with current guidelines and the Declaration of Helsinki.

### Verbal Fluency Tasks

All participants performed a German standard VF task (“RegensburgerWortflüssigkeitstest,”; Aschenbrenner et al., 2000), consisting of a semantic and a phonemic condition, each with a non-alternating and another alternating task. In the semantic VF tasks, participants were instructed to produce as many words as possible belonging to a given category. In the phonemic VF tasks, each word should begin with the same letter specified before the experiment. In the alternating VF task, participants had to alternate between two defined categories (semantic) or initial letters (phonemic). Therefore, each individual completed four VF tasks in randomized order, balanced across the two experimental sessions in the PD group. The VF tasks were (instructions given in parentheses): (i) *semantic non-alternating* (vegetables); (ii) *semantic alternating* (animals alternating with furniture); (iii) *phonemic non-alternating* (words with the initial letter S); and (iv) *phonemic alternating* (alternating between words beginning with G and R). Each VF task lasted 120 s. If necessary, utterances were disambiguated after task completion. Errors were defined and explained before testing as inappropriate words not matching the task instructions, proper names or numbers, repetitions of words or word-stems, and missed alternation if applicable. All VF tasks were digitally recorded for transcription.

#### Analysis of VF Output

We analyzed VF task performance (total word count including errors) and accuracy (error rates) for each individual VF task separately. For the analysis of word frequency, VF outputs were transcribed and the lexical class of each uttered word was defined by using a standard set of German “Part-of-speech tags” (Schmid, 1999). Word frequency of use was retrieved from the German dlexDB database^1^, see also Brysbaert et al. (2011) and Heister et al. (2011). This database is based on the German reference lexicon (“Digitales Wörterbuch der Deutschen Sprache”^2^) containing approximately 100 million entries of running words (Geyken, 2007). The normalized (the absolute number of occurrences of a given token calculated per 1 million words in the corpus) and logarithmically transformed (log10) frequencies were retrieved from the database. We used the logarithmically transformed values due to the typically skewed distribution of word frequency data, and to obtain normally distributed data for statistical analysis (Baayen, 2012). To account for words missing in the database (so-called “zero-frequencies”), we applied a Laplace transformation by adding a value of 1 to each absolute occurrence value before re-calculating the normalized and log10-transformed frequency; we did not correct the total number of tokens within the corpus given its large size and the negligible effect of this correction (Brysbaert and Diependaele, 2013).

We analyzed the change of word frequency during each individual VF task by means of a median split analysis dividing VF output into two equal parts (Juhasz et al., 2012; Woods et al., 2016). If the total number of produced words was uneven, the median word was randomly assigned to either one of the two parts. To obtain a measure least susceptible to outliers due to single words with very high frequencies (particularly in phonemic VF tasks) we calculated the median instead of the mean for each individual VF task. Furthermore, we computed the change of lexical frequency during task performance by subtracting the median word frequency of the second from the first portion for each individual VF task.

#### Statistical Analysis

VF task performance was analyzed by means of a mixed analysis of variance (ANOVA) including within-subjects factors task condition (semantic/phonemic) and task alternation (alternating/non-alternating) and between-subjects factor group. The latter included controls and the PD group either ON or OFF medication in two separate analyzes. Comparisons between medication conditions were conducted by means of a repeated measures ANOVA with the within-subjects factors task and condition as above, and medication in addition (ON/OFF). VF error rates were not normally distributed and thus compared with non-parametric tests (Wilcoxon-test for within-group comparisons, Mann-Whitney *U* test for group comparisons).

To assess the expected change of lexical frequency, we entered word frequency of parts 1 and 2 after the median split of VF output into a mixed ANOVA including within-subjects factors *decrease* (part 1/part 2), *task condition* (semantic/phonemic), and *task alternation* (alternating/non-alternating). This was done separately in the control group and the PD group in both medication conditions. Analyses of group-differences and effects of medication were conducted for the computed change between both parts of VF output. This included within-subjects factors *task condition* and *task alternation* as above and the between-subjects-factor *group* (controls/PD group); the latter included the PD group ON and OFF medication in two separate ANOVAs as above. Comparisons between medication conditions within the PD group included the within-subjects factor *medication* (ON/OFF) as in the analysis of VF task performance. In view of our main hypothesis, we only analyzed and report the main effects of *decrease, group* and *medication*, or interactions involving one of both latter factors to reduce multiple testing (Cramer et al., 2016).

Normal distribution of data was established by using the Kolmogorov-Smirnov test. For mixed ANOVAs, homogeneity of error variances was assessed by Levene’s test, homogeneity of covariances was assessed by Box’s test (for both: *p* > 0.05). For statistically significant main effects or interactions, we report F-values, degrees of freedom, p-value, and partial eta squared ($\eta_{p}^{2}$) as an estimation of the obtained effect size. The significance threshold for all statistical tests was *p* < 0.05; for *post hoc* comparisons decomposing significant interactions we report Bonferroni-corrected p-values unless for non-significant results. All statistical analyses were conducted by using the software IBM SPSS Statistics for Windows (version 27, released 2020).

## Results

### Sample Characteristics

There were no statistically significant differences of any demographic variable between both participant groups.

### VF Task Performance and Accuracy

In the following, only effects of *medication* in the PD group or differences across participant groups indicated by main effects of or interactions involving *group* are reported for brevity while omitting effects of *task condition* or *task alternation*. For an overview of task performance and accuracy in both participant groups, see Table 2.

**Table 2:** VF task performance and accuracy.

	**VF task**	**Controls**	**PD OFF**	**PD ON**
**word count**	all	23.9 (± 6.0)	20.4 (± 4.7)	21.5 (± 4.7)
	phonemic	24.9 (± 8.7)	20.8 (± 5.7)	22.9 (± 5.7)
	semantic	22.9 (± 4.7)	19.9 (± 4.7)	20.1 (± 5.1)
	phon. alternating	23.7 (± 7.5)	19.2 (± 5.2)	21.2 (± 5.3)
	phon. non-alternating	26 (± 10.5)	22.5 (± 6.8)	24.7 (± 7.1)
	sem. alternating	26.1 (± 5.2)	22.6 (± 5.6)	23.7 (± 6.8)
	sem. non-alternating	19.8 (± 5.8)	17.3 (± 5.2)	16.6 (± 4.9)
				
**error rate [%]**	all	8.9 (± 5.1)	9.8 (± 5.6)	10.9 (± 6.0)
	phonemic	6.9 (± 4.3)	8.9 (± 6.2)	11.6 (± 7.7)
	semantic	10.9 (± 7.1)	10.6 (± 7.8)	10.1 (± 6.2)
	phon. alternating	7.2 (± 6.5)	6.9 (± 7.8)	12.2 (± 11.1)
	phon. non-alternating	6.6 (± 5.8)	11 (± 7.8)	10.9 (± 7.4)
	sem. alternating	10.8 (± 7.5)	12.5 (± 10.7)	11.5 (± 7.8)
	sem. non-alternating	10.9 (± 10.8)	8.8 (± 7.5)	8.8 (± 8.0)

*Word output and error rates averaged across conditions (top rows) as well as per VF tasks. All values are the Mean (± S. D.)*.

The comparison between controls and the PD group OFF medication yielded a significant main effect of *group* (*F*_(50, 1)_ = 5.651, *p* = 0.021, $\eta_{p}^{2}$ = 0.102) with fewer words produced by the PD participants following the withdrawal of dopaminergic medication. This contrast between groups was not significant when comparing controls and the PD group ON medication (*F*_(50, 1)_ = 2.552, *p* = 0.116, $\eta_{p}^{2}$ = 0.049). In both ANOVAs, there were no significant interactions between *group* and any other variable.

When comparing ON and OFF conditions within the PD group, VF output apparently increased in the ON condition, yet the effect of *treatment* was above significance threshold (*F*_(25, 1)_ = 3.166, *p* = 0.087, $\eta_{p}^{2}$ = 0.112). There was, however, a significant interaction between *medication* and *condition* (*F*_(25, 1)_ = 6.135, *p* = 0.020, $\eta_{p}^{2}$ = 0.197). *Post hoc* comparison showed a significant increase of words produced in phonemic VF tasks in the ON compared with the OFF condition (*p* = 0.007, corrected 0.014), in contrast to semantic VF (*p* = 0.809).

A comparison of total error-rates, as well as error-rates by VF task between participant groups, did not yield significant differences between healthy volunteers and the PD group OFF medication. Error-rates were significantly increased in the PD group ON medication relative to healthy volunteers for phonemic VF only (*p* = 0.018, corrected 0.036). No significant differences in error rates emerged when comparing semantic to phonemic VF in healthy volunteers (*p* = 0.339) and PD participants OFF medication alike (*p* = 0.468). In the PD group ON medication, however, error-rates were significantly higher for phonemic than semantic VF (*p* = 0.018).

When contrasting ON and OFF conditions within the PD group, the total error-rate was significantly higher in the ON than OFF condition (*p* = 0.033). A separate analysis by VF task conditions showed that this difference mainly emerged from increased error-rates during phonemic VF in the ON condition (*p* = 0.010, corrected 0.020), whereas there was no significant difference of task accuracy in semantic VF (*p* = 0.475).

### Analysis of Lexical Frequency

**Figure 2:**
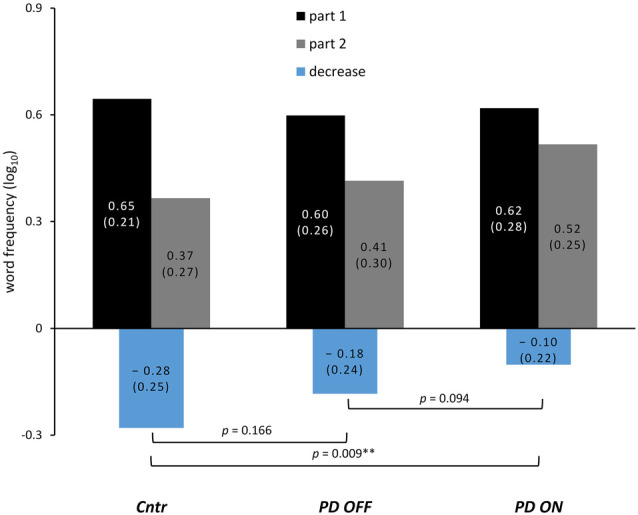
Change of word frequency during VF task performance. Lexical frequency (normalized per 1 million tokens and log_10_-transformed) of words produced during VF after the median split of word output averaged across all tasks; the change between both parts calculated by subtraction of part 1 from part 2 is shown in the bottom part. *P*-values are the main effect of the between-subjects factor *group* in the mixed ANOVAs and the within-subjects factor *medication* for the ANOVA conducted within the PD group (* for p-values < 0.05; ** for p-values < 0.01). The numbers are the mean word frequency with standard deviations in parentheses.

For the mean word frequency of VF output (averaged across all VF tasks) after median split as well as the calculated decrease, see Figure 2 and Table 3.

**Table 3:** Word frequency change across VF task performance.

**VF task**	**healthy volunteers**	**PD group medication OFF**	**PDgroup medication ON**
all	−0.280 (± 0.255)	−0.184 (± 0.237)	−0.102 (± 0.217)
phonemic	−0.245 (± 0.457)	−0.171 (± 0.431)	−0.041 (± 0.407)
semantic	−0.314 (± 0.226)	−0.196 (± 0.298)	−0.163 (± 0.261)
phon. alternating	−0.281 (± 0.584)	−0.293 (± 0.655)	0.145 (± 0.520)
phon. non-alternating	−0.208 (± 0.516)	−0.049 (± 0.572)	−0.227 (± 0.486)
sem. alternating	−0.440 (± 0.374)	−0.334 (± 0.412)	−0.322 (± 0.326)
sem. non-alternating	−0.189 (± 0.343)	−0.059 (± 0.358)	−0.004 (± 0.391)

*Decrease of word frequency during VF task performance was measured by subtraction of the mean word frequency of the second part of word output from the first part (after division in two equal portions; see also Figure 2). The decrease is, therefore, reflected by negative values. Word frequency was computed based on normalized log_10_-transformed frequency data retrieved from the dlexDB database (see “Methods” Section)*.

As expected, word frequency significantly decreased during VF task performance as indicated by a significant main effect of *decrease* in healthy controls as well as in the PD group in both medication conditions. This effect of *decrease*, however, was most pronounced in controls (*F*_(25, 1)_ = 31.141, *p* < 0.001, $\eta_{p}^{2}$ = 0.556), smaller in the PD group OFF medication (*F*_(25, 1)_ = 15.642, *p* = 0.001, $\eta_{p}^{2}$ = 0.385), and smallest in the PD group ON medication (*F*_(25, 1)_ = 5.727, *p* = 0.025, $\eta_{p}^{2}$ = 0.186).

#### Group Comparisons

The ANOVA including controls and the PD group OFF medication yielded no significant effect of *group* (*F*_(50, 1)_ = 2.449, *p* = 0.124, $\eta_{p}^{2}$ = 0.047) and no significant interaction involving *group*.

The ANOVA for controls and PD patients ON medication yielded a significant main effect of *group* (*F*_(50, 1)_ = 7.139, *p* = 0.009, $\eta_{p}^{2}$ = 0.128), indicating a smaller decrease of word frequency in the PD group ON medication relative to controls. Furthermore, the interaction of all factors *task condition*, *task alternation* and *group* was also significant (*F*_(50, 1)_ = 6.406, *p* = 0.015, $\eta_{p}^{2}$ = 0.114). To follow up on this interaction, we conducted a separate ANOVA by VF task conditions, which yielded a significant main effect of *group* for semantic VF (*F*_(50, 1)_ = 4.997, *p* = 0.03, $\eta_{p}^{2}$ = 0.091) without a significant interaction involving *group*. For phonemic VF, there was no significant effect of *group* alone (*F*_(50, 1)_ = 2.888, *p* = 0.095, $\eta_{p}^{2}$ = 0.055), but a significant interaction of *group* and *task alternation* (*F*_(50, 1)_ = 7.068, *p* = 0.011, $\eta_{p}^{2}$ = 0.124). *Post hoc* comparisons between groups revealed that the decrease of word frequency differed significantly only for the phonemic alternating VF task (*p* = 0.008) but not the non-alternating subtask of phonemic VF (*p* = 0.891).

#### Effects of Medication

The within-subjects factor *medication* did not reach statistical significance (*F*_(25, 1)_ = 3.021, *p* = 0.094, $\eta_{p}^{2}$ = 0.108). There was, however, a significant interaction of *task alternation* and *medication* (*F*_(25, 1)_ = 5.522, *p* = 0.027, $\eta_{p}^{2}$ = 0.181), as well as all three factors *task alternation*, *task condition* and *medication* (*F*_(25, 1)_ = 10.475, *p* = 0.003, $\eta_{p}^{2}$ = 0.295). A *post hoc* comparison between ON and OFF conditions of the computed decrease of word frequency for each VF task separately revealed that only phonemic alternating VF differed significantly between both medication states (*p* = 0.007, corrected *p* = 0.028).

#### Practice Effects

In order to control for possible practice effects due to the repeated testing in both medication conditions in the PD group, we repeated the latter analysis with *task condition*, *task alternation* as above as well as *session order* (first/second) instead of *medication*. This did not yield a significant main effect of *session order* on the decrease of word frequency (*F*_(25, 1)_ = 0.136, *p* = 0.716, $\eta_{p}^{2}$ = 0.005). There was a significant interaction of *task alternation* and *session order* (*F*_(25, 1)_ = 5.567, *p* = 0.026, $\eta_{p}^{2}$ = 0.182), which was due to opposite effects of session order on the decrease of word frequency in alternating and non-alternating subtasks regardless of task condition, which we omit for brevity as it does not account for the above reported contrast between medication conditions.

With respect to practice effects and group comparisons, we conducted two mixed ANOVAs for the computed decrease of word frequency with *task condition* and *task alternation* as above as well as the subjects-factor *group_2*, which included healthy controls and the PD group either at the first or second experimental session irrespective of medication status. This yielded a significant main effect of *group_2* both for the contrast controls—PD group at the first session (*F*_(50, 1)_ = 4.107, *p* = 0.048, $\eta_{p}^{2}$ = 0.076) as well as the second session (*F*_(50, 1)_ = 4.952, *p* = 0.031, $\eta_{p}^{2}$ = 0.090).

## Discussion

In this study, we analyzed the change of word frequency during VF task performance as a measure of the underlying semantic activation in participants with PD both ON and OFF dopaminergic medication relative to healthy volunteers as a control group. Our main finding is a significantly reduced decrease of lexical frequency during VF task performance in participants with PD when tested ON their regular dopaminergic medication relative to controls. This group difference was observed both during semantic and phonemic VF tasks, yet most pronounced with respect to the phonemic alternating VF subtask. The additional analysis to control for practice effects did not yield evidence for an effect of repeated testing underlying this observed contrast between healthy controls and the PD group while being ON medication.

We did not observe group-differences regarding semantic activation for the contrast between healthy controls and the PD group OFF medication that would support an aberrant pattern of semantic activation because of dopamine depletion (e.g., Arnott et al., 2001; Foster et al., 2008). Rather, our results are consistent with the assumption of a faster decay of activation in persons with PD under pharmacological treatment (Angwin et al., 2005; Castner et al., 2007a) in a similar manner as in healthy volunteers following dopamine administration (Kischka et al., 1996). The Gain/Decay hypothesis has suggested an exponential formula for modeling semantic activation patterns including a time constant determining the rise and fall of activity (Milberg et al., 1999). In view of this model, the current result would correspond to a pattern of activation, which would fall beyond a threshold of activation more rapidly as the spreading activation dissipates. The current study is the first to suggest such an effect of dopaminergic medication on semantic activation during word generation in persons with PD.

### Earlier Studies (VF)

These findings are basically consistent with the results of experiments on word priming conducted both in healthy subjects and in people with PD. However, the available data on semantic activation underlying word generation tasks are more limited (and less consistent). Most notably, our study contrasts results by Foster et al. (2008), who reported an overall lower lexical frequency of words produced during phonemic VF in a PD group ON medication (no OFF condition was included) relative to controls. This was, in light of word priming studies, interpreted as an index of *increased* semantic activation due to dopamine depletion, opposite to a reduction (i.e., smaller decay) of semantic activation found after dopamine administration. The reported group-difference, however, was not significant when using non-parametric testing, which was discussed by the authors as a possible indication of weak statistical power due to small sample sizes. Therefore, the different results obtained in our analysis could simply be attributed to larger samples, although the difference of methodological approaches appears a more likely explanation. In this regard, the analysis of the dynamic change of lexical frequency instead of the overall word frequency of all responses (which did not differ between groups or medication conditions in our study) might be a more sensitive measure of semantic activation underlying word search and retrieval. Other studies, which included lexical frequency in their analysis of semantic or phonemic VF, also reported no difference between PD groups and controls regarding the overall word frequency in semantic and phonemic VF (Herrera et al., 2012; Zabberoni et al., 2017; Wagner et al., 2020). One study, however, observed an *increased* mean word frequency of verbs produced by the PD group, which was not attributed to altered semantic activation but rather disrupted coupling between motor abilities and cognition (Herrera et al., 2012). An increase of lexical frequency in this context is rather reminiscent of lexical simplification of language due to the substitution of single words by highly frequent alternatives as observed in conditions with speech impairment (e.g., Bird et al., 2000; Cuetos et al., 2002; Boukrina et al., 2015; Faroqi-Shah and Milman, 2018; Tiedt et al., 2021). However, another study, which analyzed the dynamic change of lexical frequency during VF by means of the median split analysis in a PD group (ON and OFF medication) and healthy controls did not yield significant differences between either medication conditions or participant groups with respect to the change (i.e., decrease) of word frequency during VF tasks (Zabberoni et al., 2017). A possible explanation could be a longer duration of each individual VF task in our study (2 min as compared to one). In view of the conceptualization of low frequency words as representing distant nodes within the semantic network, a faster decay of activation might only be recognized with task durations long enough to capture this effect. This would be comparable with the observed influence of dopamine administration on word priming which was apparent only if more sustained or widespread activation was elicited by indirect word associations and longer inter-stimulus intervals (Kischka et al., 1996; Milberg et al., 1999; Angwin et al., 2004).

### Disturbed or Facilitated Lexical Access?

Of note, both connectionist models of language (Martin et al., 1994; Dell et al., 1997), as well as the Gain/Decay hypothesis (Milberg et al., 1999), have associated a faster decay of semantic activation with *disturbed* lexical access in anomic aphasia or impaired memory due to Alzheimer’s dementia (AD). In our study, however, reduced semantic activation coincided with improved VF task performance in the PD group tested ON medication, which is counterintuitive to an interpretation of this effect as indicating disrupted lexical access. On the other hand, the error rate significantly increased in the PD group ON medication, which could be taken as an argument for a negative effect in this regard. In terms of its impact on VF task performance and accuracy, we will discuss different interpretations for a faster decay of semantic spreading activation as a result of dopaminergic medication in PD in the following:

(i) A faster decay of semantic activation can be viewed as an index of an increased signal-to-noise ratio and thus more focused activity due to dopaminergic neuromodulation (Servan-Schreiber et al., 1990), cf. Kischka et al. (1996). This unspecific effect of dopamine would improve word search within lexical fields belonging to a superordinate semantic category (e.g., “pets” within the category “animals”) by facilitating the retrieval of high-frequency words. Because of weaker activation of relatively infrequent words representing distant nodes in the semantic network, more highly frequent words, which can be viewed as overlearned associations, become more easily available for retrieval (Kischka et al., 1996). Lexical alternatives with lower frequency, however, become less likely to be retrieved at the same time. This might increase the possibility of repetition errors and category errors, where semantically related or similar high-frequency words outside the demanded category are retrieved instead (e.g., fruits instead of vegetables). These effects on semantic activation by dopaminergic medication would occur in parallel with improved executive functions, particularly increased switches between lexical fields (Tiedt et al., 2020). More switches would also increase the possibility of accessing a new lexical field within a categorical network instead of a prolonged search process after retrieval of highly frequent words. This would account for the formation of smaller word clusters (Tiedt et al., 2020) and, assuming that lower frequency words reflect more distant nodes of lexical fields in analogy to the superordinate semantic network, mirror the overall effect on semantic activation at a “micro-level” (Meyer et al., 2012). The improvement of executive functions by dopamine in PD, however, can be ambiguous as it might be related to increased impulsivity and thus abnormal behaviors associated with dopaminergic medication (Weintraub et al., 2015; Voon et al., 2017). Therefore, the effects of dopamine on both semantic activation and executive functions contribute to improved task performance as well as poorer accuracy observed in the PD group ON medication. The latter can be viewed as both due to an increased rate of semantic errors and compromised task control as a result of dopaminergic medication.

(ii) A faster decay of semantic activation is by itself negative for VF performance by impeding lexical access to relatively infrequent words, yet the observed positive “net effect” of dopaminergic medication on VF performance is driven by an improvement of executive functions. This interpretation would rather follow models that view accelerated decay of semantic spreading activation as a correlate of dysfunctional network activity in conditions of disrupted lexical access (e.g., Martin et al., 1994; Dell et al., 1997; Milberg et al., 1999). In the case of this effect in PD, the impact of altered semantic activation might be rather subtle and simply outweighed by the overall positive effect of dopaminergic restoration therapy on executive functions and switching in particular (Cools, 2006). This effect could be dose-dependent and account for detrimental effects of dopaminergic overstimulation on cognition and behavior, which becomes apparent with an increased error-rate. This would imply a gradual change with an individual “tipping point” at which the negative effects on VF performance might outweigh the benefit of dopaminergic medication. The observed increase of errors in the ON condition, therefore, reflects a trade-off between improved switching and worse task-control on the other hand (Cools et al., 2003).

(iii) The observed accelerated decay of semantic activation is, with respect to VF task performance, a by-product or epiphenomenon without any apparent impact on word retrieval of its own. Therefore, the improvement of task performance, as well as poorer task control, observed between ON and OFF conditions completely rely on dopaminergic modulation of executive functions as outlined above.

In favor of both latter arguments, the direct comparison between ON and OFF conditions did not reach statistical significance, suggesting that the observed effect on semantic activation indeed is at least rather subtle and only showed up in group-comparisons between participants with PD and healthy volunteers. In comparison, improved switching during VF tasks (reflecting the modulation of executive functions) appears to be a more robust effect of pharmacological or DBS treatment based on intra-individual contrasts (Vonberg et al., 2016; Tiedt et al., 2020).

Finally, the above-discussed interpretation of our results is based on the view of frequency effects as “genuinely lexical in nature” (Jescheniak and Levelt, 1994, p. 839), and that the observed change in the decrease of word frequency occurs in addition to modulation of executive functions. However, it might be considered that the suggested dopaminergic effect on word frequency is itself a reflection of executive and particularly attentional task processing. For instance, the selection of any given word necessitates the inhibition of multiple competing semantically similar alternatives or subordinate meanings in case of lexical ambiguity. Such lexico-semantic inhibition has been shown to be impaired in PD (Castner et al., 2007b; Copland et al., 2009), so that the observed result would concur with improved lexical switching, i.e., positive effects of dopaminergic treatment in the executive domain. Furthermore, attentional capacities are required during word generation and have been most consistently associated with the lexical stage of word production (Ferreira and Pashler, 2002), which is also the locus of frequency effects (Dell, 1990; Jescheniak and Levelt, 1994; Caramazza, 1997). However, central attention is mostly involved if there is some interference between word retrieval and other cognitive demands (Roelofs and Piai, 2011), which is particularly the case in phonemic and alternating VF tasks. In PD, poor performance in alternating VF tasks has been associated with both impaired attentional control and set-shifting abilities (Zec et al., 1999). Given that effects of medication on the change of word frequency were most pronounced in the phonemic alternating VF subtask, altered allocation of attentional resources in PD may have contributed to our current result as well. At this point, the determination of the exact relationship between executive functions and lexical processes warrants further research, for instance, by a combined analysis of word frequency effects with dual-task paradigms.

### Dissociated Effects of Dopamine

A “paradoxical” effect of dopamine as discussed above (i.e., improvement of some cognitive functions as well as worsened task accuracy), would be in line with previous findings suggesting dissociated effects of the modulation of fronto-striatal and overdosing of meso-cortical (prefrontal) dopaminergic transmission on the other hand (Cools et al., 2001a). For instance, dopaminergic medication was shown to either improve or deteriorate performance on working memory tasks depending on individual baseline dopamine levels (Cools and D’Esposito, 2011). Furthermore, Cools suggested that in early PD dopamine restores impaired cognitive functions associated with dopamine depletion of the dorsal striatum whereas ventral striatal overdosing of less affected dopaminergic circuitry causes the observed detrimental effects (Cools, 2006). This view has been supported by a study using fMRI that demonstrated an increase of activity within the dorsomedial striatum associated with improved task switching in PD patients after receiving dopaminergic medication. In contrast, the increase of reward-related ventromedial striatal BOLD-signals was related to impaired reward anticipation in the same patients (Aarts et al., 2014). With respect to the time-course of dopamine depletion in PD, in earlier to medium stages of the disease dopaminergic medication causes frontal hyperactivation in less affected brain regions (Kim et al., 2018). The differential impact of STN-DBS and dopaminergic medication on the formation of word clusters (Vonberg et al., 2016; Tiedt et al., 2020) is also consistent with the supposed link between prefrontal dopaminergic (over-) stimulation and the observed faster decay of semantic activation in the current study.

### Limitations and Conclusion

Finally, some methodological considerations should be noted. The suggested effect of dopaminergic medication on semantic activation was only observed in comparisons between the PD group and healthy controls, whereas the direct comparison between both medication conditions (ON vs. OFF) did not reach statistical significance. Most likely, this is due to a rather subtle effect of dopamine on semantic activation as discussed above; however, it also warrants a cautious interpretation of the results. Furthermore, investigations following drug withdrawal in PD patients have implicated that longer washout phases might be required to obtain OFF states (Hauser et al., 2000). This might be reflected in individual cases showing only little or no motor change between ON and OFF conditions in the current study. Furthermore, as can be taken from Figure 2 and Table 3, the decrease of word frequency appeared to be smaller in the PD group OFF medication as well (relative to controls). This difference is only suggested and not statistically significant, yet it might as well indicate an incomplete washout of dopaminergic medication. A longer withdrawal of dopaminergic medication, however, would increase the strain on the participants beyond an acceptable level. In our study, several participants could not sustain withdrawal from dopaminergic medication, which resulted in a slightly unbalanced order of the two experimental sessions. As VF assessments, like other neuropsychological tests, have been shown to exhibit practice effects regarding word counts particularly in phonemic VF (Bartels et al., 2010), this should be balanced more rigorously in future studies. The additional analysis of practice effects, however, argues against repeated testing as an explanation of the current results: the comparison between healthy controls and the PD group categorized by session order instead of medication status significantly reduced or leveled the contrast between participant groups. In case of practice effects underlying the observed difference between controls and the PD group ON medication, it should have been increased instead. Finally, the analysis of lexical frequency as based on corpus data collected from written texts such as books or newspaper articles has to be viewed as an approximation to the frequency of use during spoken language. A different approach that might be of interest in future studies has been suggested with the construction of databases drawn from film subtitles (e.g., New et al., 2007).

To conclude, we found that dopaminergic medication is associated with a faster decay of semantic spreading activation underlying VF task performance in participants with PD relative to healthy volunteers. As this observation went along with an improvement of VF task performance in PD patients, a faster decay of semantic activation would be consistent with an increase of the signal-to-noise ratio by dopamine resulting in more focused neural activity. In view of the effects of dopamine on executive functions, the observed improvement of word generation, as well as increased error-rates ON medication, suggest a “trade-off” between task performance and accuracy, consistent with dissociated effects of dopamine restoration at the fronto-striatal level and possible overdosing of meso-cortical dopaminergic circuits. Future studies could aim to relate intra-individual motor fluctuations and dyskinesia as markers of dopaminergic over-stimulation (Voon et al., 2009) to subjectively experienced cognitive changes during these states in particular.

## Data Availability Statement

The raw data supporting the conclusions of this article will be made available by the authors, without undue reservation.

## Ethics Statement

The studies involving human participants were reviewed and approved by Charité—Universitätsmedizin Berlin (EA2/047/10). The patients/participants provided their written informed consent to participate in this study.

## Author Contributions

HT conceptualized the study, contributed to data acquisition, analyzed the data, and wrote the manuscript. FE contributed to the conceptualization of the study, data acquisition, and revised the draft of the manuscript. FK contributed to the conceptualization and critically revised the manuscript draft. All authors contributed to the article and approved the submitted version.

## Conflict of Interest

The authors declare that the research was conducted in the absence of any commercial or financial relationships that could be construed as a potential conflict of interest.

## Publisher’s Note

All claims expressed in this article are solely those of the authors and do not necessarily represent those of their affiliated organizations, or those of the publisher, the editors and the reviewers. Any product that may be evaluated in this article, or claim that may be made by its manufacturer, is not guaranteed or endorsed by the publisher.

## Footnotes

^1^ http://www.dlexdb.de

^2^ https://www.dwds.de
